# Prevalence of hepatic steatosis as assessed by controlled attenuation parameter (CAP) in subjects with metabolic risk factors in primary care. A population-based study

**DOI:** 10.1371/journal.pone.0200656

**Published:** 2018-09-18

**Authors:** Núria Fabrellas, Rosario Hernández, Isabel Graupera, Elsa Solà, Pilar Ramos, Natividad Martín, Gemma Sáez, Consuelo Simón, Almudena Pérez, Teresa Graell, Andrea Larrañaga, Manel Garcia, Ana de la Arada, Adrià Juanola, Alicia Coiduras, Isabel Duaso, Angel Casado, Julian Martin, Marta Ginès, Nuria Moreno, Ana Gema Perez, Laia Marti, Mireia Bernat, Montse Sola, Carmina Olivé, Cristina Solé, Pere Ginès

**Affiliations:** 1 School of Medicine and Health Sciences, University of Barcelona, Barcelona, Catalonia, Spain; 2 Institut d’Investigacions Biomèdiques August Pi i Sunyer (IDIBAPS), Barcelona, Catalonia, Spain; 3 Centro de Investigación Biomédica en Red de Enfermedades Hepáticas y Digestivas (CIBEREHD), Barcelona, Catalonia, Spain; 4 Centre d’Assistència Primària La Marina, Institut Catalá de la Salut (ICS), Barcelona, Catalonia, Spain; 5 Liver Unit, Hospital Clínic, University of Barcelona, Barcelona, Catalonia, Spain; Medizinische Fakultat der RWTH Aachen, GERMANY

## Abstract

**Background:**

Primary care is the ideal setting for early identification of patients with non-alcoholic fatty liver disease (NAFLD). NAFLD is a potentially progressive disease that may lead to cirrhosis and liver cancer but is frequently underrecognized because subjects at risk are often not evaluated. Controlled attenuation parameter (CAP) is a reliable method for non-invasive quantification of liver fat. It has the advantage of simultaneous measurement of liver stiffness (LS), an estimate of liver fibrosis. There is no information on CAP in subjects with risk factors from primary care.

**Aim:**

To investigate the prevalence of hepatic steatosis, as estimated by CAP, in subjects from the community with metabolic risk factors and correlate findings with clinical and biochemical characteristics and LS.

**Patients and methods:**

Population-based study of 215 subjects with metabolic risk factors without known liver disease identified randomly from a primary care center. A control group of 80 subjects matched by age and sex without metabolic risk factors was also studied. CAP and LS were assessed using Fibroscan.

**Results:**

Subjects with risk factors had CAP values higher than those of control group (268±64 vs 243±49dB/m,p<0.001). Prevalence of severe steatosis (CAP> 280dB/m) in subjects with risk factors was 43%. In multivariate analysis, fatty liver index (FLI) and HOMA were independent predictive factors of severe steatosis. There was a direct correlation between CAP and FLI values (r = 0.52,p<0.001). Interestingly, prevalence of increased LS was 12.6% in the risk group vs 0% in the control group (p<0.001). Increased LS occurred predominantly in subjects with high CAP values.

**Conclusions:**

A high proportion of subjects with metabolic risk factors seen in primary care have severe steatosis. FLI could be used as a surrogate of CAP. Increased LS was found in a significant proportion of subjects with risk factors but not in control subjects.

## Introduction

Non-alcoholic fatty liver disease (NAFLD) is a major health problem worldwide because of its high prevalence and its important long-term morbidity and mortality [1–5]. NAFLD affects approximately 25% of the population worldwide and its incidence is growing rapidly because of associated metabolic comorbidities, such as obesity, type-2 diabetes, hyperlipidemia, and metabolic syndrome, the frequency of which is increasing at a very fast rate in most areas of the world [1–3]. The presence of fat in the liver is associated with an increased risk of liver-related morbidity and mortality through development of liver fibrosis and cirrhosis [6,7]. Moreover, patients with NAFLD have decreased survival compared to that of the general population due to cardiovascular complications, development of cirrhosis, and hepatic as well as non-hepatic tumors [5,8,9].

The diagnosis of NAFLD relies on the demonstration of presence of hepatic steatosis in the absence of secondary causes of fat accumulation such as significant alcohol consumption, use of certain drugs or hereditary disorders [10,11]. The ideal setting for the diagnosis of NAFLD is primary care because patients with risk factors for NAFLD are usually seen in the community by primary physicians or nurse practitioners caring for their metabolic comorbidities. The most commonly used method for diagnosis of hepatic steatosis in the community is liver ultrasonography because it is simple and widely available. However, liver ultrasonography has several drawbacks, particularly limited sensitivity, difficulty in the morbidly obese, it is operator dependent, only provides qualitative or semi-quantitative information about the amount of fat, and is not useful for the detection of concomitant liver fibrosis [12]). In NAFLD, liver fibrosis is important because its presence and severity predicts cirrhosis development and long-term survival [9,13,14]. Other methods to estimate the amount of fat in the liver such as proton magnetic spectroscopy or serum biomarkers, such as fatty liver index (FLI), Steatotest^R^, and NAFLD fibrosis score, are generally not used in primary care [11,12].

Controlled attenuation parameter (CAP) is a system that measures the degree of ultrasound attenuation by hepatic fat using a process based on vibration control transient elastography [12]). A number of studies have demonstrated that CAP has high predictive accuracy of the amount of fat in the liver, particularly in patients with NAFLD [15–17]. Therefore, CAP is currently considered a precise noninvasive method for assessment of hepatic fat. Moreover, CAP has the additional advantage of the simultaneous evaluation of liver fibrosis by measurement of liver stiffness (LS). [12]

Although there are numerous studies evaluating CAP in large series of patients with NAFLD in tertiary hospitals, there is little information on the application of CAP in primary care [18]. Particularly, there is no information on the use of CAP in assessment of hepatic steatosis in subjects with metabolic risk factors in the community. In the current study, we used CAP to assess the prevalence of hepatic steatosis in patients with metabolic risk factors identified randomly in a primary care center. The prevalence was compared to that of a control group of subjects without metabolic risk factors. CAP values were correlated with clinical and biochemical variables and also with LS.

## Patients and methods

### Aims

The current study was aimed at investigating the prevalence of steatosis as assessed by CAP in subjects from the community setting with metabolic risk factors but without known liver disease. Secondary objectives were: 1) to compare the prevalence of steatosis in subjects with metabolic risk factors with that of a control group of similar age and sex without metabolic risk factors; and 2/ to correlate CAP values with clinical and biochemical characteristics as well as LS.

### Population and study protocol

**Fig 1:**
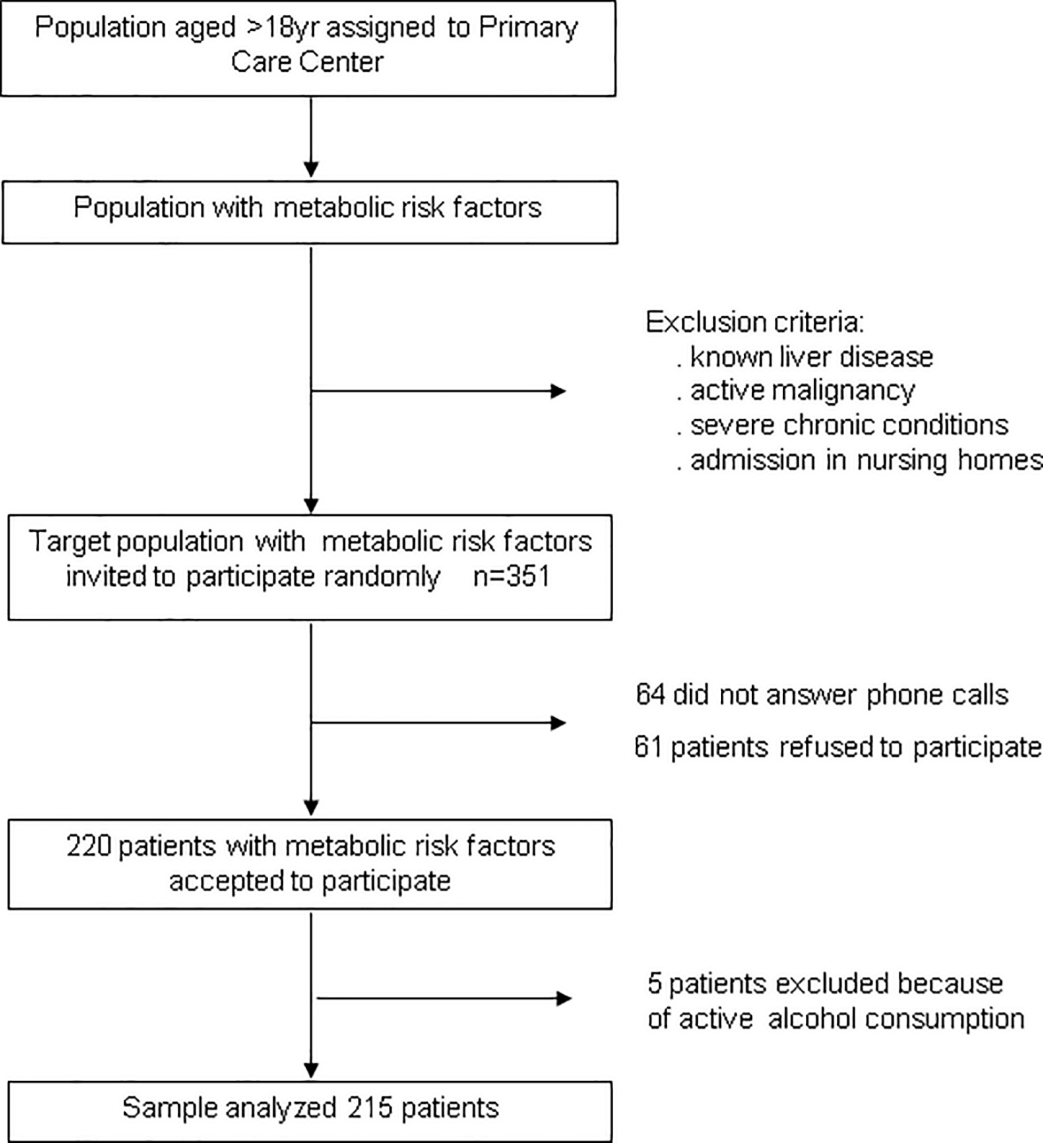
The flow chart of the study.

This is a population-based, cross-sectional study that included 215 subjects with metabolic risk factors but without know liver disease from the community setting. Subjects were recruited from primary care center La Marina (Barcelona) and considered eligible for participation in the study if they had at least one of the following metabolic risk factors, as reported elsewhere [10]: 1/ obesity; 2/ type-2 diabetes mellitus; 3/ dyslipidemia; and 4/ metabolic syndrome, as defined by presence of 3 or more of the following features: a/ waist circumference greater than 102 cm in men or 88 cm in women; b/ serum triglycerides ≥150 mg/dL; c/ high-density lipoprotein (HDL) cholesterol levels less than 40 mg/dL in men or less than 50 mg/dL in women; d/ systolic blood pressure ≥130 mmHg or diastolic blood pressure ≥85 mmHg; and e/ fasting plasma glucose ≥110 mg/dL [19]. Subjects aged >18yr were identified using computer-generated random numbers from the patient registry that contains clinical information of citizens assigned to the primary care center using metabolic risk factors shown above as keywords. Patients with known diagnosis of liver disease were excluded. Other exclusion criteria were active malignancy, severe chronic conditions, and admission in nursing homes. Eligible subjects were then contacted by telephone by nurses or general practitioners from the primary care center and invited to participate in the study. Subjects interested were invited to attend the primary care center where a member of the research team explained carefully the objectives of the investigation and the study protocol (Fig 1).

Patients who accepted signed a written informed consent. At the visit in the primary care center, demographic and clinical data were collected from all participants. Weight, height, waist circumference, and arterial pressure were measured. In addition, a blood sample was taken to determine standard liver tests, lipid profile, serum ferritin, serum creatinine, glycated hemoglobin, and fasting insulin levels. FLI was calculated using standard formula [20]. In all subjects, CAP and LS were measured (see later). Subjects with LS >7kPa with the M probe or >6.2 kPa with the XL probe, were referred to the Liver Unit of the Hospital Clínic of Barcelona for hepatology consultation, which consisted of assessment of liver disease following a diagnostic protocol that included disease assessment and staging with liver tests, liver ultrasonography, and liver biopsy in patients who accepted the procedure. Subjects with LS <7 or 6.2 with the M and XL probes, respectively, did not undergo further evaluation. These cutoffs were selected on the basis of those used in previous studies [21–23]. A control group of subjects matched (2:1) by sex and age (±5 years) with those of the study group, but without metabolic risk factors was studied for comparison. Subjects for the control group were selected among subjects attending consultations of primary care physicians. Of the 110 subjects who accepted to participate, 30 subjects were subsequently excluded because metabolic risk factors were identified during study assessment (n = 24) or alcohol risk consumption (n = 6). Therefore, the control group consisted of 80 subjects. The protocol was approved by the Investigational Review Boards of the primary care center (Fundació Gol i Gorina) and Hospital Clínic i Provincial of Barcelona.

### CAP and liver stiffness measurements

CAP and LS measurements were performed by a single experienced operator (PR) using Fibroscan system 502 touch^R^ (Echosens, Paris). Either M or XL probes were used. The decision to use the M or XL probes was made following the recommendation of the software of the system. The principles of CAP have been reported in detail elsewhere [12,24]. Measurements were always performed in the morning after overnight fast. CAP was computed only when LS measurement was valid and with the same signals used to measure LS. The final CAP value was the median of individual CAP values and was expressed in dB/m. In 3 of the 295 subjects included (1%), LS could not be determined (body mass index–BMI- and waist circumference in these patients were 43, 35, and 34 Kg/m2 and 140, 111, and 114 cm, respectively). The levels of CAP used to define the presence and degree of steatosis were as follows: 1/ <248dB/m, no steatosis (S0); 2/ 248–268 dB/m, mild steatosis (S1); 3/ >268 and ≤280 dB/m, moderate steatosis (S2); and 4/ >280 dB/m, severe steatosis (S3), as reported elsewhere [25]. Reliability of LS measurements using specific criteria [26] showed that all measurements performed were either reliable or very reliable (65% and 35%, respectively). No measurements had poor realiability using these criteria.

### Statistical analysis

Results for continuous variables were expressed as mean and standard deviation. Counts and percentages were used for the description of the categorical variables. Comparisons between two independent groups were made with the t-test (previously checking the hypothesis of variance homogeneity) for continuous normal-distributed variables. The Mann-Whitney U test was carried out for continuous non-normal distributed variables in the case of 2 independent groups. Comparison between variables of more than 2 groups was performed with ANOVA or Kruskal-Wallis. Comparisons of categorical variables among groups were made with chi-squared test or Fisher test if appropriate. Transaminase levels have been considered classically as surrogate markers of steatosis. Therefore, the predictive accuracy of FLI in the diagnosis of severe steatosis was compared with that of AST/ALT levels using AUROC curves.

Multivariate logistic regression models were performed to assess independent predictive factors of steatosis, severe steatosis, and LS. The significance level for all statistical tests was set at 0.05 two-tailed. All statistical analyses were performed using SPSS 20.0 software.

## Results

### Characteristics of the study population

Comparison of the demographic, clinical, and biochemical characteristics of subjects from the risk group and control group is shown in Table 1.

**Table 1:** Comparison of demographic, clinical, and biochemical data, and liver stiffness and CAP of subjects from the risk group and control group.

Variable	Risk group n = 215	Control group n = 80	P value
Age (yr)	58 ± 12	62 ± 13	0.03
Male gender	35 (44%)	91 842%)	0.9
Tobacco consumption	45 (20%)	17 (19%)	0.7
Diabetes Mellitus	60 (28%)	-	<0.001
Dyslipidemia	150 (70%)	-	<0.001
Obesity (BMI≥30 Kg/m2)	113 (52%)	-	<0.001
Metabolic syndrome	98 (46%)	-	<0.001
**Number of risk factors**			
**1**	86 (40%)	-	<0.001
**2**	66 (31%)	-	
**≥3**	63 (29%)	-	
BMI (Kg/m^2^)	31 ± 5	25 ± 2	<0.001
Waist circumference (cm)	104 ± 13	91 ± 10	<0.001
Glucose (mg/dL)	112 ± 40	89 ± 10	<0.001
Total cholesterol (mg/dL)	204 ± 44	198 ± 26	0.15
HDL- cholesterol (mg/dL)	49 ± 14	58 ± 16	<0.001
LDL-cholesterol(mg/dL)	129 ± 33	123± 24	<0.001
Triglycerides (mg/dL)	138 ± 83	83 ± 24	<0.001
ALT (IU/L)	26 ± 16	24 ± 11	0.16
GGT (IU/L)	34 ± 35	27 ± 40	0.16
Serum creatinine (mg/dL)	0.7 ± 0.1	0.7 ± 0.2	0.6
Albumin (g/L)	44 ± 2	43 ± 2	0.8
Ferritin (ng/mL)	125 ± 137	104 ± 91	0.21
Glycated hemoglobin (%)	6.2 ± 1	5.5 ± 0.3	<0.001
HOMA	5.7 ± 8.6	2.2 ± 1.4	<0.001
LS (kPa)	4.9 ± 2.7	4.2 ± 0.9	0.002
CAP (dB/m)	268 ± 64	243 ± 49	0.001

Values are number and percentages (in brackets) or mean±SD. BMI, body mass index; ALT, alanine aminotransferase; GGT, gamma glutamyltranspeptidase; HOMA, Homeostasis model assessment; LS, liver stiffness; CAP, controlled attenuation parameter

As expected, due to the inclusion criteria, subjects with metabolic risk factors had marked alteration of laboratory variables, such as glucose, triglycerides, HDL-cholesterol, glycated hemoglobin, and HOMA, compared to control subjects without metabolic risk factors.

### Prevalence of steatosis and factors associated with CAP values

The degree of steatosis and LS were related to the presence of metabolic risk factors. Subjects with metabolic risk factors had significantly higher CAP and LS values compared to those of control subjects (268±64 vs 243±49 dB/m and 4.9±2.7 vs 4.2±0.9 kPa, respectively; p<0.01 for both) (Table 1). Moreover, the prevalence of steatosis was significantly higher in the risk factor group compared to the control group, regardless the cutoff values of CAP used for steatosis grading (Table 2).

**Table 2:** Prevalence of steatosis in patients with (risk group) and without risk factors for NAFLD (control groups).

	Risk group n = 215	Control group n = 80	P value
CAP > 248dB/m (S0 vs S1-2-3)	136 (63%)	37 (42%)	0.01
CAP > 268dB/m (S0-1 vs S2-3)	106 (49%)	27 (34%)	0.02
CAP > 280dB/m (S0-1-2 vs S-3)	93 (43%)	24 (30%)	0.045

Values are numbers of subjects and percentages (in brackets)

Tables 3 to 5 show the comparison of baseline characteristics of subjects categorized into different subgroups according to the cutoff levels of CAP of 248, 268, and 280 dB/m, which allows a comparison of subjects with S0 vs S1-2-3 (Table 3), S0-1 vs S2-3 (Table 4), and S-0-1-2 vsS3 (Table 5).

**Table 3:** Comparison of baseline characteristics of subjects with risk factors for NAFLD categorized according to the presence (S1-2-3) or absence (S0) of steatosis measured by CAP values.

Variable	CAP < 248dB/m S0	CAP ≥ 248dB/m S1-2-3	
	n = 79	n = 136	
Age (yr)	63 ± 13	62 ± 12	0.7
Male gender	30 (38%)	61 (45%)	0.4
Tobacco consumption	12 (15%)	31 (23%)	0.4
Diabetes Mellitus	16 (20%)	44 (32%)	0.06
Dyslipidemia	53 (67%)	97 (71%)	0.54
Obesity (BMI≥30kg/m^2^)	30 (38%)	83 (61%)	0.002
Metabolic syndrome	28 (35%)	70 (51%)	0.02
**Number of risk factors**			
**1**	14 (18%)	15 (11%)	0.03
**2**	29 (38%)	45 (34%)	
**>3**	28	70 (51%)	
BMI (Kg/m^2^)	29 ± 5	32 ± 5	<0.001
Waist circumference (cm)	100 ± 12	106 ± 12	<0.001
Glucose (mg/dL)	102 ± 35	118 ± 42	0.006
Total cholesterol (mg/dL)	203 ± 46	204 ± 43	0.9
HDL- cholesterol (mg/dL)	53 ± 17	47 ± 12	0.008
LDL-cholesterol(mg/dL)	129 ± 36	128 ± 31	0.9
Triglycerides (mg/dL)	113 ± 69	152 ± 87	0.001
ALT (IU/L)	22 ± 11	28 ± 17	0.002
GGT (IU/L)	29 ± 30	38 ± 38	0.08
Creatinine (mg/dL)	0.7 ± 0.3	0.7 ± 0.2	0.7
Albumin (g/L)	44 ± 2	44 ± 3	0.8
Ferritin (ng/mL)	111 ± 109	134 ± 151	0.2
Glycated hemoglobin (%)	5.9 ± 0.9	6.3 ± 1.1	0.01
HOMA	3.5 ± 2.8	7.0 ± 10.4	<0.001
FLI	52 ± 27	73 ± 21	<0.001
LS (kPa)	4.2 ± 1.1	5.3 ± 3.2	<0.001
CAP (dB/m)	204 ± 38	305 ± 43	<0.001

Values are number and percentages (in brackets) or mean±SD. BMI, body mass index; ALT, alanine aminotransferase; GGT, gamma glutamyltranspeptidase; HOMA, Homeostasis model assessment; LS, liver stiffness; CAP, controlled attenuation parameter.

**Table 4:** Comparison of baseline characteristics of subjects with risk factors for NAFLD categorized according to the presence (S2-3) or absence (S0-1) of moderate-severe steatosis measured by CAP values.

Variable	CAP < 268dB/m S0-1	CAP ≥ 268dB/m S2-3	
	n = 109	n = 106	
Age (yr)	62 ± 13	63 ± 12	0.5
Male gender	42 (38%)	49 (46%)	0.27
Tobacco consumption	20 (18%)	23 (22%)	0.46
Diabetes Mellitus	20 (18%)	40 (38%)	0.002
Dyslipidemia	72 (66%)	78 (74%)	0.24
Obesity (BMI≥30kg/m^2^)	43 (40%)	70 (66%)	<0.001
Metabolic syndrome	38 (35%)	60 (57%)	0.002
**Number of risk factors**			
**1**	18 (17%)	11 (11%)	0.001
**2**	42 (40%)	32 (31%)	
**>3**	38 (35%)	60 (56%)	
BMI (Kg/m^2^)	29 ± 5	33 ± 5	<0.001
Waist circumference (cm)	100 ± 12	107 ± 12	<0.001
Glucose (mg/dL)	101 ± 31	124 ± 45	<0.001
Total cholesterol (mg/dL)	205 ± 44	202 ± 45	0.54
HDL- cholesterol (mg/dL)	52 ± 16	46 ± 11	<0.001
LDL-cholesterol(mg/dL)	129 ± 34	128 ± 32	0.79
Triglycerides (mg/dL)	119 ± 70	157 ± 91	0.001
ALT (IU/L)	23 ± 11	30 ± 18	0.001
GGT (IU/L)	28 ± 27	41 ± 42	0.009
Creatinine (mg/dL)	0.7 ± 0.2	0.8 ± 0.2	0.6
Albumin (g/L)	44 ± 25	44 ± 2.6	0.9
Ferritin (ng/mL)	108 ± 1.17	142 ± 154	0.08
Glycated hemoglobin (%)	5.9 ± 0.7	6.5 ± 1.2	<0.001
HOMA	3.4 ± 2.6	8.5 ± 11	<0.001
FLI	54 ± 26	77 ± 20	<0.001
LS (kPa)	4.3 ± 1.2	5.6 ± 3.6	0.001
CAP (dB/m)	218 ± 40	319 ± 38	<0.001

Values are number and percentages (in brackets) or mean±SD. BMI, body mass index; ALT, alanine aminotransferase; GGT, gamma glutamyltranspeptidase; HOMA, Homeostasis model assessment; LS, liver stiffness; CAP, controlled attenuation parameter

**Table 5:** Comparison of baseline characteristics of subjects with risk factors for NAFLD categorized according to the presence (S3) or absence (S0-1-2) of severe steatosis measured by CAP values.

Variable	CAP < 280dB/m S0-1-2	CAP ≥ 280dB/m S3	
	n = 122	n = 93	
Age (yr)	62 ± 13	63 ± 12	0.5
Male gender	48 (39%)	43 (46%)	0.3
Tobacco consumption	24 (19%)	19 (21%)	0.63
Diabetes Mellitus	25 (21%)	35 (38%)	0.009
Dyslipidemia	83 (68%)	67 (72%)	0.55
Obesity (BMI≥30kg/m^2^)	49 (41%)	64 (69%)	<0.001
Metabolic syndrome	46 (38%)	52 (56%)	0.009
**Number of risk factors**			
**1**	21 (18%)	8 (9%)	0.003
**2**	44 (37%)	30 (33%)	
**>3**	46 (38%)	52 (56%)	
BMI (Kg/m^2^)	29 ± 5	33 ± 5	<0.001
Waist circumference (cm)	100 ± 11	109 ± 11	<0.001
Glucose (mg/dL)	103 ± 36	124 ± 43	<0.001
Total cholesterol (mg/dL)	207 ± 45	199 ± 43	0.18
HDL- cholesterol (mg/dL)	52 ± 16	45 ± 11	<0.001
LDL-cholesterol(mg/dL)	130 ± 34	126 ± 32	0.37
Triglycerides (mg/dL)	122 ± 69	159 ± 94	0.001
ALT (IU/L)	23 ± 12	30 ± 19	0.006
GGT (IU/L)	29 ± 28	41 ± 43	0.02
Creatinine (mg/dL)	0.7 ± 0.2	0.8 ± 0.2	0.6
Albumin (g/L)	44 ± 2	44 ± 2	0.9
Ferritin (ng/mL)	109 ± 114	146 ± 161	0.05
Glycated hemoglobin (%)	5.9 ± 0.9	6.4 ± 1.1	0.001
HOMA	3.7 ± 3.3	8.2 ± 12	0.001
FLI	56 ± 25	78 ± 20	<0.001
LS (kPa)	4.4 ± 1.2	5.6 ± 3.8	<0.001
CAP (dB/m)	224 ± 41	325 ± 36	<0.001

Values are number and percentages (in brackets) or mean±SD. BMI, body mass index; ALT, alanine aminotransferase; GGT, gamma glutamyltranspeptidase; HOMA, Homeostasis model assessment; LS, liver stiffness; CAP, controlled attenuation parameter

**Fig 2:**
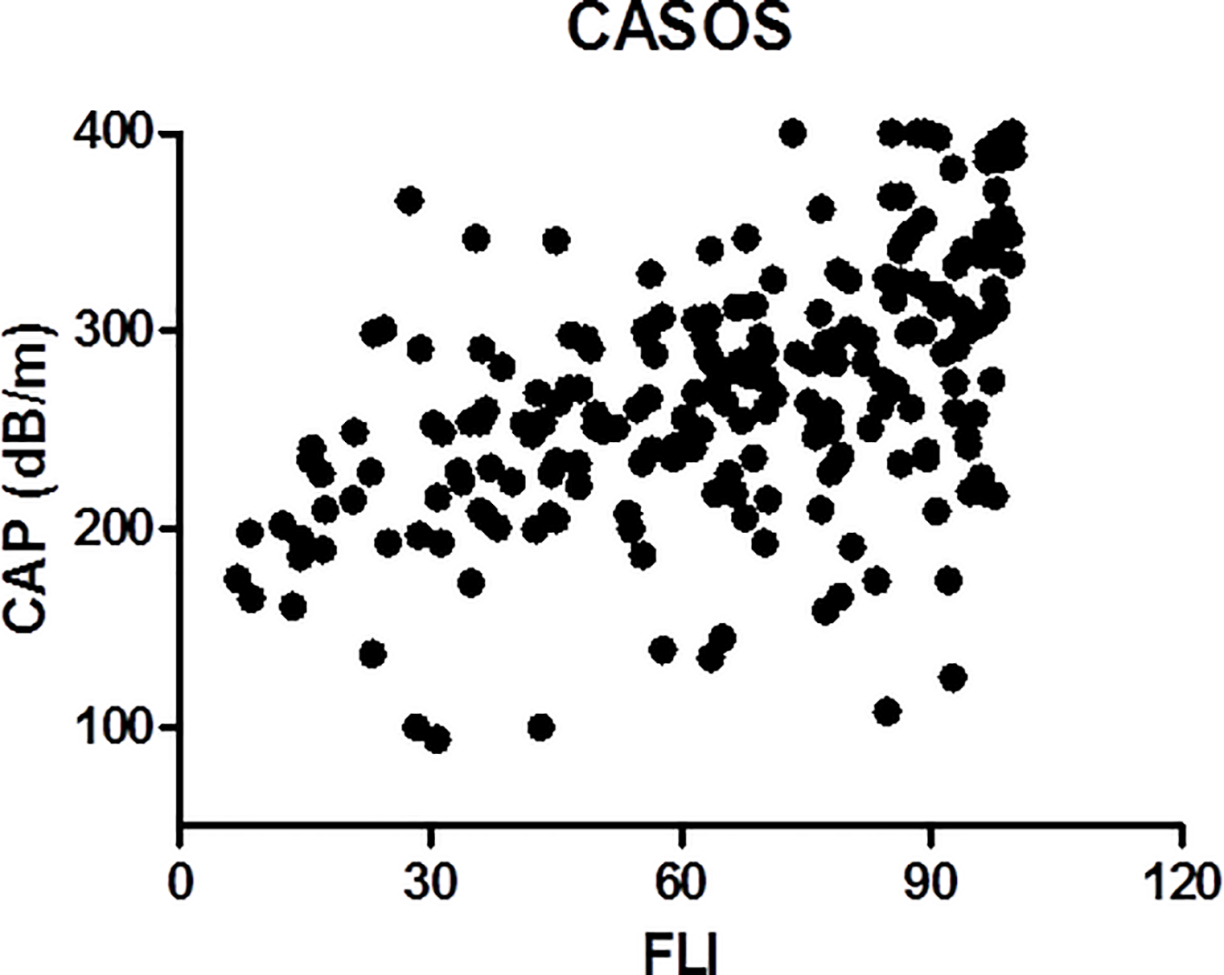
Correlation between CAP and FLI values in the 215 subjects with metabolic risk factors of NAFLD included in the study.

The degree of steatosis was associated with diabetes mellitus, obesity, metabolic syndrome, greater BMI and waist circumference, and higher glucose, triglycerides, ALT, GGT, ferritin, glycosylated hemoglobin, and HOMA levels, and lower HDL-cholesterol. Moreover, the degree of steatosis was also associated with higher FLI values. There was a statistically significant direct correlation between CAP and FLI values (r = 0.52, p<0.001) (Fig 2).

In multivariate analysis, factors independently associated with steatosis or severe steatosis were FLI alone or in association with HOMA, respectively (Table 6).

**Table 6:** Multivariate Logistic Regression analysis of variables associated with steatosis and severe steatosis.

Steatosis (CAP ≥ 248dB/m)			
Independent Variable	OR	CI	P value
FLI	1.032	1.019–1.046	<0.01
**Severe steatosis (CAP ≥ 280dB/m)**			
**Independent Variables**			
FLI	1.036	1.02–1.052	<0.001
HOMA	1.085	1.007–1.169	0.031

Variables included in the model for Steatosis: FLI, Triglycerides, BMI, Waist circumference, HDL-cholesterol, ALT, Glycated Hb and HOMA.

Variables included in the Severe Steatosis model: FLI, Diabetes, BMI,Waist circumference, HDL-cholesterol, ALT, Glycated Hb and HOMA

If FLI was not included in the multivariate analysis, variables independently associated with CAP>248 dB/m were triglycerides and BMI, and those associated with CAP>280 dB/m were triglycerides, waist circumference, and glycated hemoglobin. The direct relationship between CAP and FLI was also observed in the whole population of subjects included in the study, with and without metabolic risk factors (r = 0.48, p<0.001).

**Fig 3:**
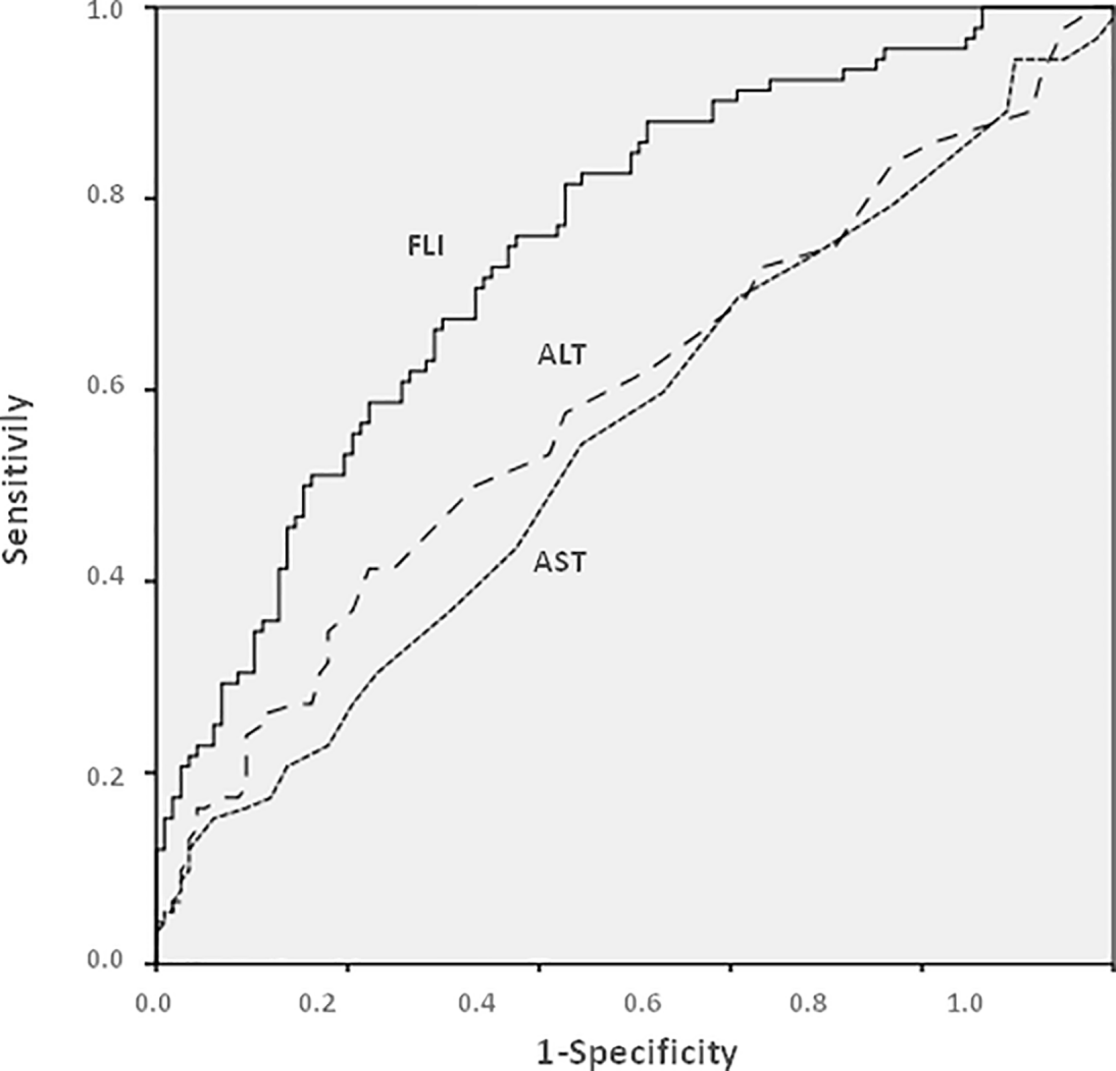
Predictive accuracy of FLI, AST and ALT, as assessed by AUROC curves, in the diagnosis of severe steatosis in the group of patients with metabolic risk factors of NAFLD.

As shown in Fig 3, the predictive accuracy of FLI in the diagnosis of severe steatosis was significantly better than that of AST or ALT.

The best cutoff value of FLI for the diagnosis of severe steatosis was of 96. Similar findings were observed when the whole population of subjects, with and without risk factors, was considered or when analyzing moderate/severe steatosis instead of severe steatosis (data not shown). It is important to emphasize that 84 of the 106 (79%) subjects with risk factors and moderate or severe steatosis had normal AST and ALT values.

### Liver stiffness measurement and relationship with CAP

Twenty-seven of the 215 subjects (12.6%) of the risk group had increased LS, as estimated by values greater than 7kPa or 6.2 kPa, with the M and XL probes, respectively. By contrast, none of the 80 subjects from the control group without metabolic risk factors had increased LS (x^2^ = 11.1; p<0.001). If a higher cutoff of LS was considered (>8Kpa) the prevalence of increased LS in the risk group was of 4% (9 of the 215 subjects). Factors associated with increased LS in univariate analysis were diabetes mellitus, obesity, BMI, waist circumference, glucose, ALT, glycated hemoglobin, HOMA, and CAP (Table 7)

**Table 7:** Comparison of characteristics of subjects with risk factors for NAFLD categorized according to liver stiffness.

Variable	Normal LS N = 188	Increased LS* N = 27	P value
Age	62 ± 12	65 ± 13	0.2
Male gender	76 (83%)	15 (16%)	0.15
Diabetes Mellitus	46 (24%)	14 (52%)	0.005
Dyslipidemia	135 (72%)	15 (56%)	0.12
Obesity (BMI≥30kg/m^2^)	94 (50%)	19 (73%)	0.03
Metabolic syndrome	83 (44%)	15 (56%)	0.3
BMI (Kg/m^2^)	30 ± 5	35 ± 6	<0.001
Waist circumference (cm)	102 ± 11	115 ± 13	<0.001
Glucose (mg/dL)	107 ± 34	146 ± 58	0.002
Total cholesterol (mg/dL)	206 ± 45	188 ± 36	0.05
HDL- cholesterol (mg/dL)	50 ± 15	46 ± 13	0.2
LDL-cholesterol(mg/dL)	130 ± 34	118 ± 28	0.1
Triglycerides (mg/dL)	134 ± 80	165 ± 97	0.065
ALT (IU/L)	25 ± 13	36 ± 24	0.02
GGT (IU/L)	31 ± 28	57 ± 69	0.06
Albumin (g/L)	44 ± 2	43 ± 3	0.6
Ferritin (ng/mL)	115 ± 109	194 ± 253	0.1
Glycated hemoglobin (%)	6 ± 0.9	6.8 ± 1.3	0.007
HOMA	4.7 ± 6.2	12.6 ± 16.61	0.03
FLI	63 ± 25	85 ± 18	<0.001
CAP (dB/m)	261 ± 59	316 ± 71	<0.001

*>7 Kpa with M probe or >6.2 kPa with XL probe.

Values are number and percentages (in brackets) or mean±SD. BMI, body mass index; ALT, alanine aminotransferase; GGT, gamma glutamyltranspeptidase; HOMA, Homeostasis model assessment; LS, liver stiffness; CAP, controlled attenuation parameter.

In multivariate analysis, variables independently associated with increased LS were diabetes mellitus, waist circumference, and ALT (Table 8).

**Table 8:** Multivariate logistic regression analysis of variables associated with increased liver stiffness in subjects with risk factors for NAFLD.

VARIABLE	OR	CI	P value
Diabetes Mellitus	4.8	1.7–13.4	0.002
Waist circumference	1.1	1.04–1.1	<0.001
ALT	1.03	1.003–1.057	0.03

Variables included in the model: BMI, waist circumference, ALT, Glycated Hb and HOMA, Diabetes mellitus, CAP, FLI

**Fig 4:**
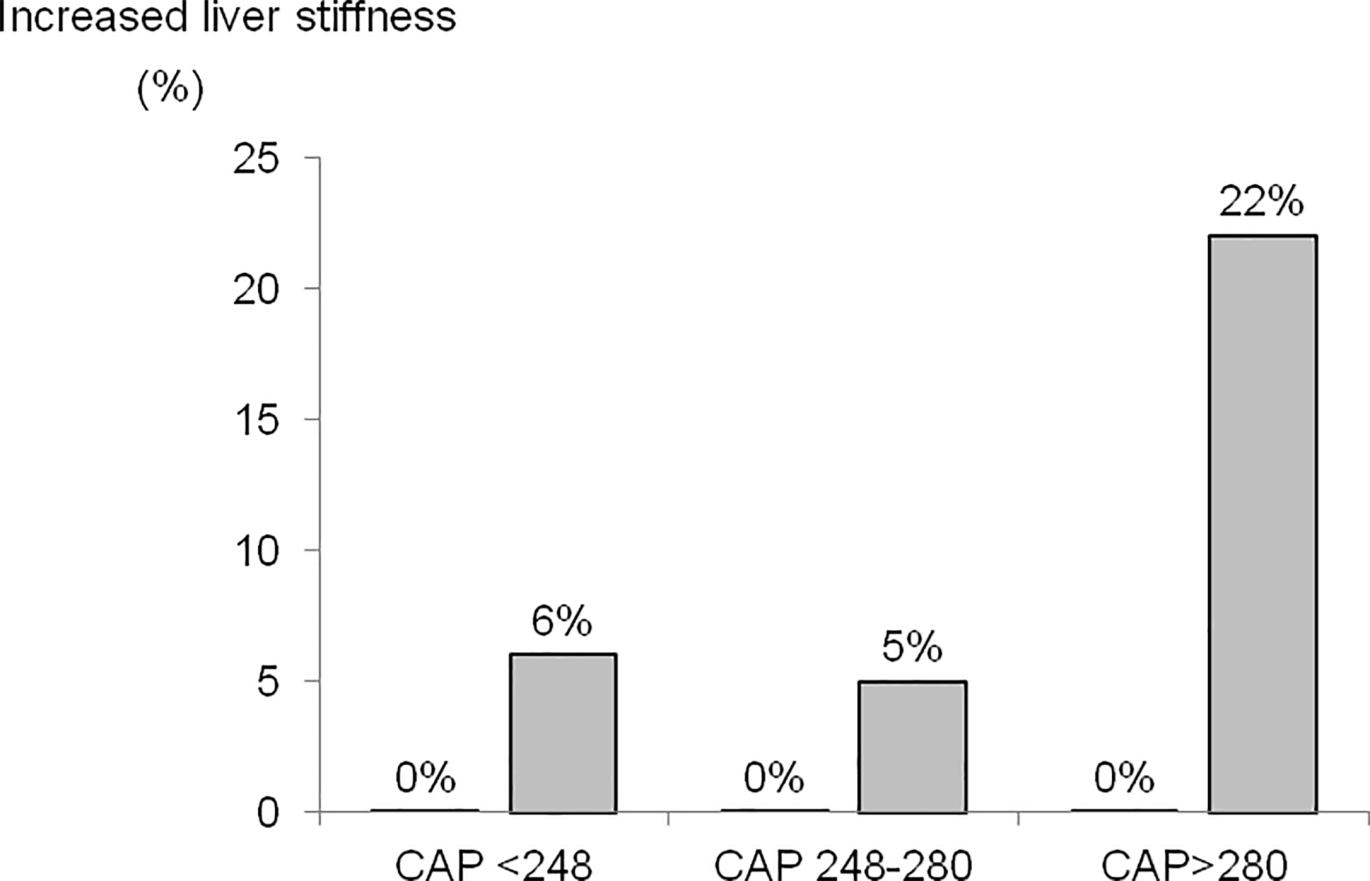
Prevalence of increased liver stiffness (>7kPa or >6.2kPa with the M and XL probes, respectively), in the group of subjects with risk factors for NAFLD (grey bars) and control group (no bars because of 0% prevalence) categorized in 3 subgroups according to CAP values.

Prevalence of increased LS was significantly greater in subjects with severe steatosis (S3) than in those with mild/moderate (S1-2) or no steatosis (S0) (22% vs 5 and 6%, respectively (Fig 4). Remarkably, in the control group without metabolic risk factors, none of the subjects had increased LS regardless of CAP values (Fig 4).

**Fig 5:**
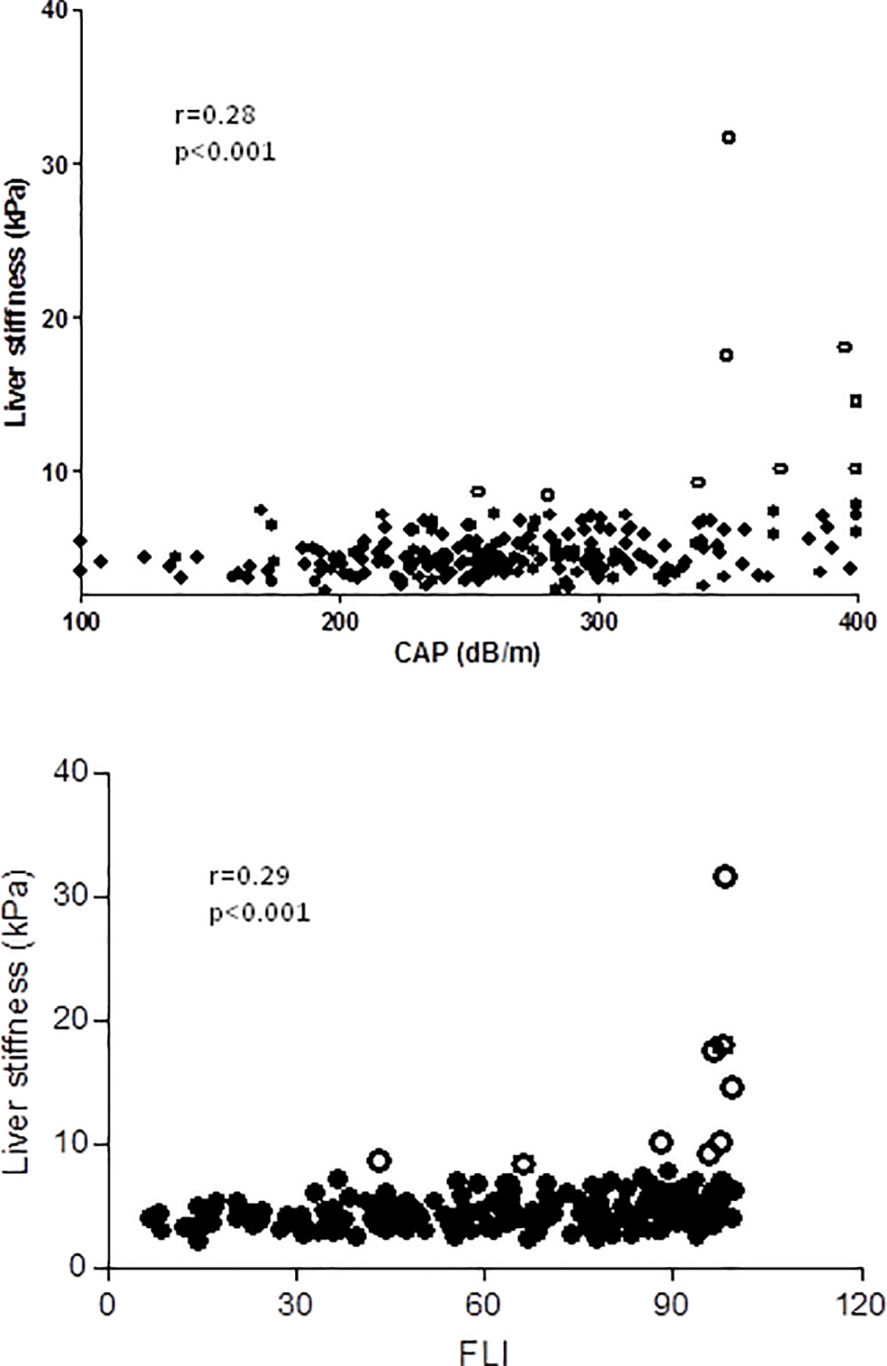
(upper panel) Relationship between liver stiffness and CAP values in patients with risk factors for NAFLD. (lower panel) Relationship between liver stiffness and FLI values in patients with risk factors of NAFLD. Empty circles represent patients with liver stiffness greater than 8 kPa.

Fig 5 shows the relationship between LS and CAP and LS and FLI in subjects with metabolic risk.

The 27 patients with increased LS were referred to the hospital for hepatology consultation and 22 accepted. In all 22 patients the diagnosis of NAFLD was confirmed by ultrasonography. Mean LS and CAP values in these patients were 10.0±5.9kPa (range 6.6–31.6) and 323±67 dBm (range 170–400), respectively. Nine of the 22 patients (40%) underwent a liver biopsy. LS values in patients in whom a liver biopsy was performed were 13.3±8.2 kPa (range 7–31.6). Histological examination showed significant liver fibrosis in 3 (F2, F3 and F4) associated with moderate steatosis, and steatosis without fibrosis in 6 patients (severe and moderate in one patient each, and mild in the remaining 4 patients).

## Discussion

The results of the current study show that a high proportion of subjects with metabolic risk factors from the community, without known liver disease, have severe steatosis as indicated by high values of CAP. The degree of steatosis did not correlate with transaminase levels but showed good correlation with FLI. Increased LS, suggestive of liver fibrosis, was only found in subject with metabolic risk factors and increased CAP values.

Epidemiological studies indicate that NAFLD affects more than 25% of the adult population worldwide and is particularly common among subjects with metabolic risk factors [1–3]. Patients with NAFLD should be identified not only because they are at risk of developing liver fibrosis subsequently leading to cirrhosis and hepatocellular carcinoma, but also because they have increased mortality due to cardiovascular events [1–3]. Although hepatic steatosis without inflammation has been classically considered a “benign” condition, without risk of progression, recent studies indicate that as much as one fourth of patients with simple steatosis can progress to steatohepatits and fibrosis within a short period of time [27,28]. Most subjects at risk for NAFLD are in the community setting and not in the hospital unless they develop some acute complications. Therefore, identification should ideally be performed in primary care. Interestingly, it has been reported that NAFLD is frequently underrecognized in the primary care setting and subjects with metabolic risk factors are not frequently evaluated for this condition [29,30].

Along these lines, the results of the current study, in which CAP was used for evaluation of hepatic steatosis, demonstrate that 43% of adults subjects with metabolic risk factors and previously unrecognized liver disease identified randomly from primary care have severe steatosis. Previous studies have shown that other noninvasive methods to estimate hepatic steatosis, including hepatic ultrasound or transaminase levels, lack sensitivity in the detection of hepatic steatosis [12]. CAP is not a perfect method, but studies in which CAP values have been compared with liver histology, show that the AUROC curves for severe steatosis (>66% of hepatocytes) are greater than 0.8 [16]. Therefore, it is likely that the majority of subjects identified in the current study with high CAP values had severe steatosis. Unfortunately, histological confirmation is not available because liver biopsy was only performed in a small number of subjects who had increased LS. As expected from results of previous studies, CAP values correlated strongly with metabolic factors, particularly diabetes mellitus, obesity, metabolic syndrome, BMI, waist circumference, glucose, LDL-cholesterol, triglycerides, ALT, glycated hemoglobin, and HOMA [15–18].

Another interesting observation of the current study was the existence of a direct correlation between CAP and FLI values. Remarkably, in multivariate analysis, FLI was an independent predictive factor of steatosis and severe steatosis. This strong direct correlation found is probably related to the fact that the four components of FLI (BMI, waist circumference, triglycerides, and GGT) are strongly related to metabolic syndrome [20]. Our findings confirm previous observations in subjects from the general population [18]. Altogether, these findings suggest that FLI can be used as a surrogate marker to estimate hepatic steatosis in subjects in the community setting if the determination of CAP is not available.

Our study also evaluated the relationship between CAP and LS. The correlation between CAP values and LS was very weak, both in the group of subjects with metabolic risk factors and in the overall population of subjects. In this context, it is important to remind that our population was composed of subjects from the community setting in which, contrarily to that of the hospital setting, the prevalence of increased LS is low. However, a closer look at individual values of CAP and LS showed that increased LS was almost exclusively observed in patients with high CAP values indicative of severe steatosis. Of interest, none of the subjects from the control group had increased LS despite the fact that some of them had relatively high CAP values, yet lower than those in the metabolic risk group. The number of subjects with histological examination was relatively low. In some subjects, significant liver fibrosis was confirmed (from F2 to F4), whereas in others there was only steatosis without significant liver fibrosis. This lack of fibrosis in some subjects may be related to the fact that steatosis “per se” may increase LS values [31]. The possibility of a sampling error in liver biopsy also exists yet it is difficult to prove. Recent studies have demonstrated that the presence of steatosis increases LS and therefore the cut-off level for significant liver fibrosis in patients with NAFLD should be around 9kPa, higher than that in other disease states such as viral hepatitis [23,32]. The relationship between LS and CAP values has been investigated in great detail in a recent study by Karlas et al which analyzed a very large population of patients with different etiologies of chronic liver disease and from different geographical areas [33]. Overall, the study showed that CAP has a small impact on classification of patients with significant fibrosis according to LS. In populations with low prevalence, consideration of CAP can improve slightly the already high negative predictive value of LS in ruling out significant liver fibrosis. Moreover, the study confirmed that the accuracy of LS for detecting significant liver fibrosis is limited, due to high rate of false-positive values, particularly in patients with large CAP values [33]. As an example, in our study 3 subjects who underwent a liver biopsy for LS > 14 kPa and who had CAP values ≥350 dB/m did not have fibrosis in the liver biopsy.

The findings of the current study also provide interesting information with respect to potential screening strategies for liver fibrosis related to NAFLD in primary care [34,35]. Our results support that screening should be focused in subjects with metabolic risk factors and that subjects without metabolic risk factors should not be screened because of very low probability of significant liver fibrosis. In fact, none of the subjects without metabolic risk factors included in the study had increased LS. Interestingly, a recent large population-based study showed that the prevalence of LS>9.2kPa among subjects without risk factors was of only 0.4%, compared to 5.4% in subjects with risk factors [23].

The present study has several strengths: 1/ it is population-based; patients were randomly selected from subjects attending a primary care center; 2/ both the M and XL probes of the Fibroscan system were used; this allowed acquiring reliable measurements in the majority of subjects included in the study despite a high proportion of obese patients; and 3/ a control group of subjects without metabolic risk factors was evaluated for comparison. However, the study has also some limitations that should be mentioned: 1/ liver histology should ideally have been obtained in a higher proportion of patients to correlate histological findings with CAP values; however, although this may be feasible in series of patients from hospital care, this is unrealistic in the setting of primary care, where the acceptability of invasive procedures is very low in relatively healthy populations; 2/ the study was performed in a single primary care center of an urban area; therefore, results would require validation in other primary care centers, also from non-urban areas; and 3/ the sample size is relatively low and results should ideally be validated in larger subject populations.

In conclusion, almost half of subjects with metabolic risk factors with unknown liver disease status seen in primary care have increased values of CAP, indicative of moderate-to-severe steatosis. FLI could be used as a surrogate marker of CAP in primary care because of good correlation between the two variables. Significant liver fibrosis was only found in subjects with metabolic risk factors and associated high CAP values, yet the frequency of liver fibrosis found was lower compared to that in previous studies.
